# Arthroscopic Coracoclavicular Reconstruction Combined with Open Acromioclavicular Reconstruction Using Knot Hiding Clavicular Implants Is a Stable Solution

**DOI:** 10.1016/j.asmr.2021.08.002

**Published:** 2021-08-27

**Authors:** Juha O. Ranne, Severi O. Salonen, Terho U. Kainonen, Jussi A. Kosola, Lasse L. Lempainen, Mika T. Siitonen, Pekka T. Niemi

**Affiliations:** a Hospital Mehilainen Neo, Turku, Finland, Turku, Finland; b The Paavo Nurmi Centre, Department of Physical Activity and Health, the University of Turku, Turku, Finland; c Department of Orthopaedics and Traumatology, Helsinki University Hospital, Helsinki, Finland; d Mectalent Medical Services Oy, Oulu, Finland

## Abstract

**Purpose:**

The purpose of this noninterventional, register-based study was to report the outcomes and wound healing of surgically treated chronic acromioclavicular (AC) dislocations using a tendon graft and knot-hiding titanium implants.

**Methods:**

Thirty-two cases with chronic AC separation underwent an arthroscopic coracoclavicular (CC) ligament reconstruction and an open AC ligament reconstruction using knot-hiding titanium implants. The wound healing was assessed 2 months after the operation. The Nottingham Clavicle score, Constant score, and Simple Shoulder Test were obtained postoperatively and at a minimum of one-year postsurgery. The radiographic change in distance between the clavicular and coracoid cortices and clavicular tunnel diameter was measured. General patient satisfaction with the outcome (poor, fair, good, or excellent) was also assessed 1 year postoperatively.

**Results:**

The mean Nottingham Clavicle score increased from a preoperative mean of 41.66 ± 9.86 to 96.831 ± 5.86 (*P* ≤ .05). The Constant score increased from a preoperative mean of 44.66 ± 12.54 to 93.59 ± 7.01 (*P* ≤ .05). The Simple Shoulder Test score increased from a preoperative mean of 7.00 ± 2.14 to 11.84 ± .63 (*P* ≤ .05). The coracoclavicular distance increased from 11.32 ± 3.71 to 13.48 ± 3.79 mm (*P* ≤ .05). The clavicular drill hole diameter increased from 6 mm to a mean of 6 to a mean of 8.13 ± 1.12 mm. Twenty-three (71.9%) patients reported an excellent outcome, and nine (28.1%) reported a good outcome. One clavicular fracture occurred but no coracoid fractures. There was one reconstruction failure leading to a reoperation.

**Conclusions:**

In this series, combining the arthroscopic CC ligament reconstruction to an open reconstruction of the AC joint with a tendon graft proved to be a stable solution. The knot-hiding titanium implant effectively eliminated the problems related to the clavicular wound healing.

**Level of Evidence:**

Level IV, therapeutic case series.

## Introduction

Acromioclavicular (AC) dislocation is a common injury and frequently occurs when a person falls and lands on his/her shoulder.^1^ Numerous techniques have been proposed for surgical treatment of AC separation. However, no consensus regarding treatment has been reached. For lower-grade injuries, the treatment is conservative. In Rockwood grade III injuries, the AC and coracoclavicular (CC) ligaments are completely torn, and the distal clavicle appears elevated, although the upper extremity actually returns to its normal positions. In grade V injuries, attachments of the surrounding muscle insertions on the clavicle are also ruptured.^2^^,^^3^ In grade IV injuries, the distal clavicle is posteriorly displaced into the trapezius muscle, while in type VI injuries, the dislocation is underneath the coracoid process. Most grade III separations are treated conservatively, although some of them may remain painful and unstable and eventually need surgical treatment. Grades IV–VI injuries usually undergo operative treatment.^4^^,^^5^ The original open techniques included temporary fixations with screws, pins, and plates, but arthroscopy-assisted techniques using tendon grafts have recently emerged. Previous arthroscopic techniques included washers, buttons, and interference screws for graft fixation. According to the latest reports and meta-analysis reconstructions, using a tendon graft gives the best results in chronic AC separations.^6^^,^^7^ The complication rates in surgical treatment can be quite high and appear to be related to reconstruction failure, clavicular or coracoid fracture, and/or infections.^8^, ^9^, ^10^, ^11^ The foreign material may also induce wound irritations and persistent palpable resistances underneath the skin on the clavicle.^12^

The purpose of this noninterventional, register-based study was to report the outcomes and wound healing of surgically treated chronic AC dislocations using a tendon graft and knot-hiding titanium implants. The hypothesis was that the arthroscopic CC ligament reconstruction combined with open AC ligament reconstruction would be associated with favorable clinical and radiographic results, and the knot-hiding clavicular implant would enhance clavicular wound healing.

## Methods

Patients with chronic Rockwood grades III and V AC dislocations, which were treated using two different modifications of the surgical technique as described by Ranne et al., were identified in a patient registry.^13^^,^^14^ The study was approved by the Institutional Research Board. The patients were selected prior to surgery and included individuals who were committed to following the postsurgical instructions. The injury was graded III if the coracoclavicular and acromioclavicular ligaments were torn and V if in addition, the surrounding muscle insertions were detached from the clavicle, and the distal clavicle appeared to be severely elevated. Cases needing other major surgeries, such as rotator cuff repair, were excluded. All patients underwent surgery via the same arthroscopic techniques by two senior surgeons in Hospital Mehilainen Neo. The method was chosen by the surgeons on a case-by-case basis. The knot-hiding clavicular clip designed to be used with a tendon graft was used in all cases. Clavicular wound healing and early-onset complications were followed. The Nottingham Clavicle score, Constant score, and Simple Shoulder Test were obtained to measure the outcomes. Similarly, the radiographic change in distance between the clavicular and coracoid cortices and clavicular tunnel diameter was measured. General patient satisfaction with the outcome (poor, fair, good, or excellent) was also assessed.

### Surgical Technique

**Fig 1:**
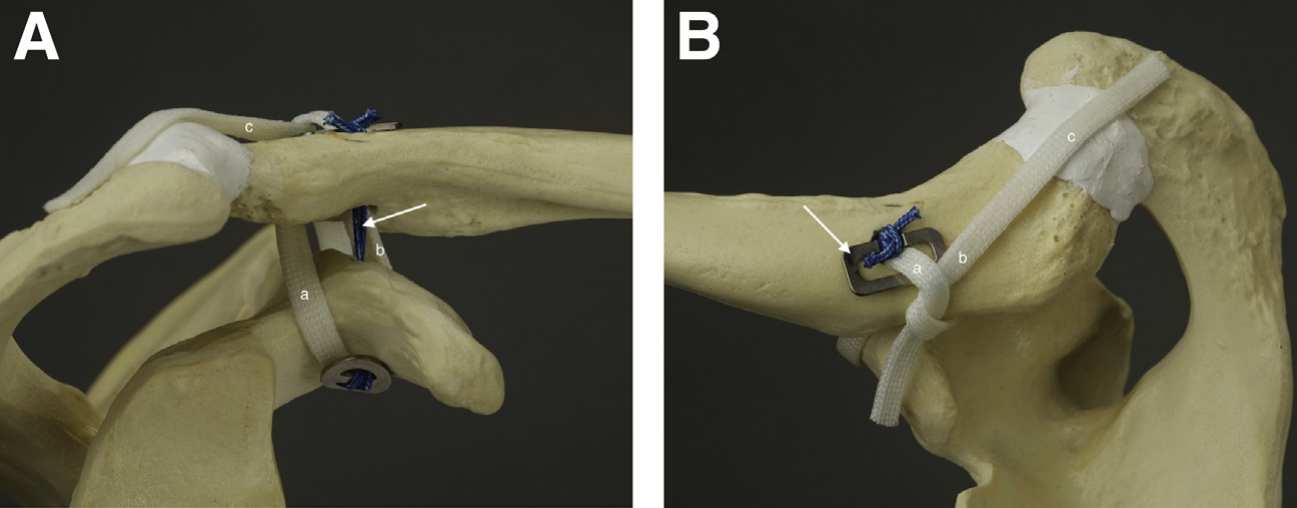
(A) The anterior graft limb (a) replaces the trapezoid ligament, while the dorsal limb (b) replaces the conoid ligament. The end of the dorsal tendon graft (b) is taken over the acromioclavicular (AC) joint recreating the superior AC ligament (c). The interconnecting suture (arrow). Anterior view of the reconstruction at which the tendon graft circles the coracoid. An anterior view, right shoulder. (B) The anterior graft limb comes through the clavicular drill hole (a). The dorsal graft limb is wrapped around the dorsal edge of the clavicle (b). The dorsal graft limb is left longer and taken over the AC joint (c). The knot-hiding clavicular clip (arrow). A superior view, right shoulder.

The technique involved an arthroscopic double-bundle reconstruction of the CC ligament complex and an open reconstruction of the AC ligaments using a semitendinosus (ST) autograft. The synthetic polyurethane-urea tendon graft (Artelon, Atlanta, GA) was used in cases in which no semitendinosus autograft was available. In the CC reconstruction, the anterior graft limb projected superiorly and replaced the trapezoid ligament, while the dorsal limb of the graft was wrapped around the dorsal edge of the clavicle and reconstructed the conoid ligament. The dorsal end of the tendon graft was taken over the AC joint to reconstruct the superior AC ligament and capsule (Fig 1). These procedures led to stabilization of the AC joint and reduction of the anterioposterior movement of the distal clavicle. A supportive semitemporary fixation of the reconstruction was achieved by connecting the Clavicular Clip (CC-Clip Turku, Finland) to the Subcoracoid Clip (CC-Clip, Turku, Finland) using a strong doubled no. 5 nonabsorbable suture. The tendon graft shared the same drill holes with the supportive fixation. Because only one centrally positioned 6-mm drill hole on the clavicle was present, the risk of fracture was small. The tendon graft could be attached to the coracoid in two manners. Either the graft was taken through the coracoid or it countered the coracoid.

The patient was placed in the beach chair position. A standard 30° arthroscope was used. The technique included five aspects (posterior, lateral, anterolateral anterior, and clavicular). Arthroscopy was initiated by inserting the arthroscope into the joint through the posterior portal. The lateral portal was opened by inserting a needle in the front of the long-head biceps tendon aimed at the proximal coracoid. The coracoid neck was exposed, and for that area, the interval was opened. After good access was established to the coracoid neck area, the arthroscope was moved to the lateral portal. The primary camera position during the actual reconstruction was in the lateral portal. The anterolateral and anterior portals were opened using a needle pointing to the coracoid neck. Debridement and sufficient exposure were conducted around the coracoid and clavicle. A longitudinal incision, 2.5 cm medially from the acromioclavicular joint, was made over the clavicle. The superior surface of the clavicle was exposed for drilling. A blunt tissue passageway was made behind the clavicle from the same opening for subsequent passing of the graft.

### Graft Passed Through the Coracoid

A 2.4-mm guide pin was driven through the clavicle and the coracoid using a drill guide after which a 4.5-mm drill hole was made through the clavicle and coracoid. The perfect position of the coracoid drill hole essentially dictated the position of the clavicular drill hole. The clavicular drill hole was centrally located on the clavicle at ∼2.5 cm proximal to the acromioclavicular joint. The clavicular drill hole was then widened to 5.5 mm. The sutures were then passed through the drill holes using the blunt Lasso Guide (CC-Clip, Turku, Finland). The tendon graft was first pulled through the clavicular and coracoid drill holes after which the suture for the interconnecting no. 5 suture was pulled through the clavicular and coracoid drill holes.

### Graft Countering the Coracoid

A 2.4-mm guide pin was driven through the clavicle and the coracoid, after which only the clavicular drill hole was widened, to 5.5 mm. The suture for the tendon graft was passed through the clavicular drill hole and then medially under the coracoid using the Curved Lasso Guide (CC-Clip, Turku, Finland) positioned in front of the clavicle and medial to the coracoid. Next, the proximal end of the lasso wire was picked up through the clavicular drill hole using a suture passer for subsequent transfer of the suture to the tendon graft. The tendon graft was then pulled through the clavicular drill hole and around the coracoid.

The rest of the surgical procedure was identical in both groups. The distal graft limb end was pulled into the clavicular wound behind the clavicle. The interconnecting sutures were passed through the drill holes and pulled out through the anterolateral portal. The Subcoracoid Clip (CC-Clip, Turku, Finland) was attached to the no. 5 suture loop and then pulled into position underneath the coracoid. The other end of the no. 5 suture and the anterior graft limb were slipped through the Clavicular Clip eyelet. The end of the dorsal graft limb was taken over the dorsal edge of the clavicle and tied to the anterior graft limb. The dorsal graft limb was kept longer than the anterior one.

**Fig 2:**
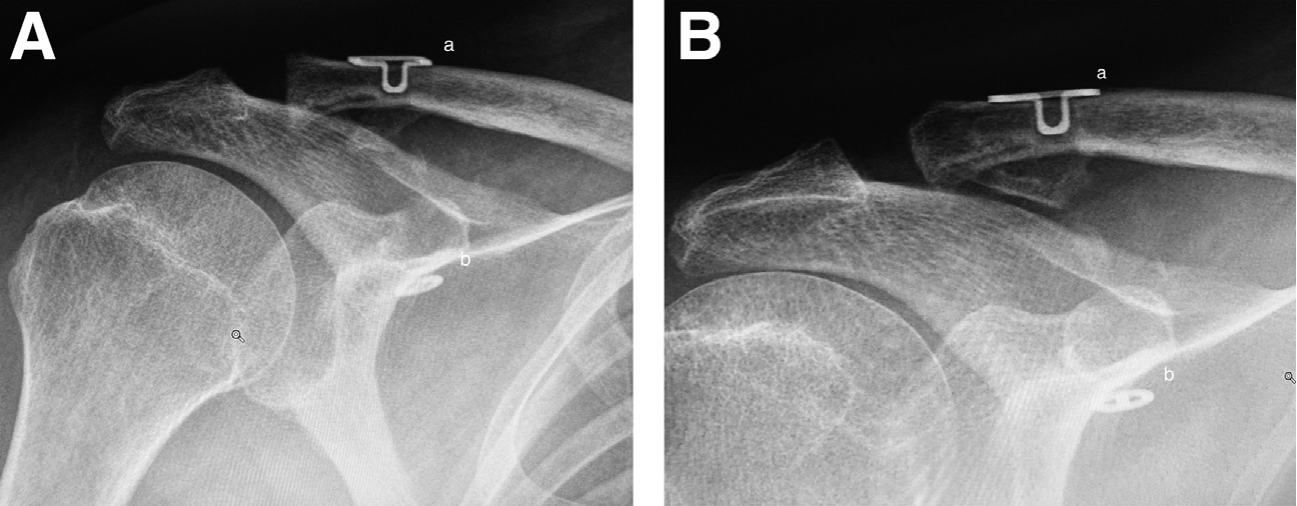
(A) A postoperative radiograph of the coracoclavicular CC reconstruction. The Clavicular Clip (a), the Subcoracoid Clip (b). Anteroposterior view, right shoulder. (B) A one-year postoperative radiograph of the CC reconstruction. The Clavicular Clip (a), the Subcoracoid Clip (b). Anteroposterior view, right shoulder.

After the actual CC reconstruction was complete, the clavicular wound was extended over the AC joint. The overstretched AC joint capsule was opened along its fibers. The distal end of the clavicle was resected with an oscillating saw, and the distal clavicle was freed from possible soft tissue attachments and scar tissue. With the entire reconstruction in place, the clavicle was repositioned. The repositioning was visually controlled and checked. The interconnecting suture and tendon graft of the CC reconstruction were tensioned, and the interconnecting sutures were tied on the CC-Clip loop using a knot pusher. Once the sutures were tied, the loop of the Clavicula Clip was allowed to sink into the clavicular drill hole hiding the suture knot. The anterior graft limb end was then tied to the posterior graft limb and secured with no. 2 nonresorbable sutures. The superior AC ligament was reconstructed with the dorsal end of the tendon graft. The graft end was sutured over the AC joint, and the AC capsule was plicated tightly over it with interrupted no. 1 resorbable sutures. The arthroscopic portals were closed with interrupted sutures, while the clavicular wound was closed in layers (Fig 2).

### Postoperative Treatment

The patients were discharged the same day as the surgery, and each wore an arm sling for six weeks postsurgery. They were allowed to perform light rotatory movements and passive arm lifting within the limits of pain. Although the sling was removed after 6 weeks, active rehabilitation did not begin until 8 weeks after surgery, to provide enough time for recovery. The patients were allowed to resume heavy labor 3 to 4 months after surgery and overhead activity and sports at 6 months.

### Statistical Analysis

Statistical analyses were calculated using the Minitab software, version 18.1 (State College, PA). This software includes descriptive univariate statistics (arithmetic mean, standard deviation), 95% confidence intervals (CI), *t*-tests, and graphs. Welch’s *t*-test was used to determine whether preoperative and postoperative groups were different from each other. For all *t*-tests, the null hypothesis stated that the difference between preoperative and postoperative groups was 0. For the clavicle drill hole diameter, significance could not be calculated because the perioperative diameter was not measured. Instead, the nominal drill diameter was used. However, in this case, a postoperative 95% CI of the mean was visually compared to the perioperative drill diameter to verify a statistically significant increase in the drill hole diameter. Regarding variables other than the drill hole diameter, statistical significance was declared when *P* < .05.

## Results

A total of 32 consecutive patients were included. Nine (28.1%) of the cases were graded III and 23 (71.8%) were graded V, of which three had a lateral clavicular fracture. The time from original trauma to surgery varied from 4 weeks to 3 years. Twenty-nine (90.6%) operations were primary cases, while three (9.4%) were revision cases that followed previous failed surgeries. Twenty-nine operations (90.6%) were conducted using a semitendinosus tendon graft, while three (9.4%) were conducted with a synthetic polyurethane urea graft.^16^

Nineteen (59.4%) of the cases underwent surgery with the tendon graft going through the coracoid drill hole (Group 1), while 13 (40.6%) were treated with the graft loop going under the coracoid (Group 2). Thirty (93.8%) cases were male, whereas two were (6.3%) female. The mean age of the patients was 41.16 ± 12.93 years. In nineteen of the cases (58.3%), the dominant side underwent surgery.

Indications for surgery included pain, distal clavicle instability, and scapular issues. Injuries older than 2 weeks were considered chronic.^15^ The clavicular wound healing and early-onset complications were followed until 2 months after surgery. The Nottingham clavicle score, Constant score, and Simple Shoulder Test were taken postoperatively and minimum of 1 year after surgery. Similarly, an anteroposterior radiograph was taken postoperatively and one year after surgery. The change in the distance between the clavicular and coracoid cortex and clavicular tunnel diameter was measured 1 year after surgery. General patient satisfaction with the outcome (poor, fair, good, and excellent) was also assessed 1 year postoperatively.

No wound infections related to clavicular wounds were found. No complaints about protruding knots on the clavicle or any sensation of foreign material underneath the skin were reported.

**Fig 3:**
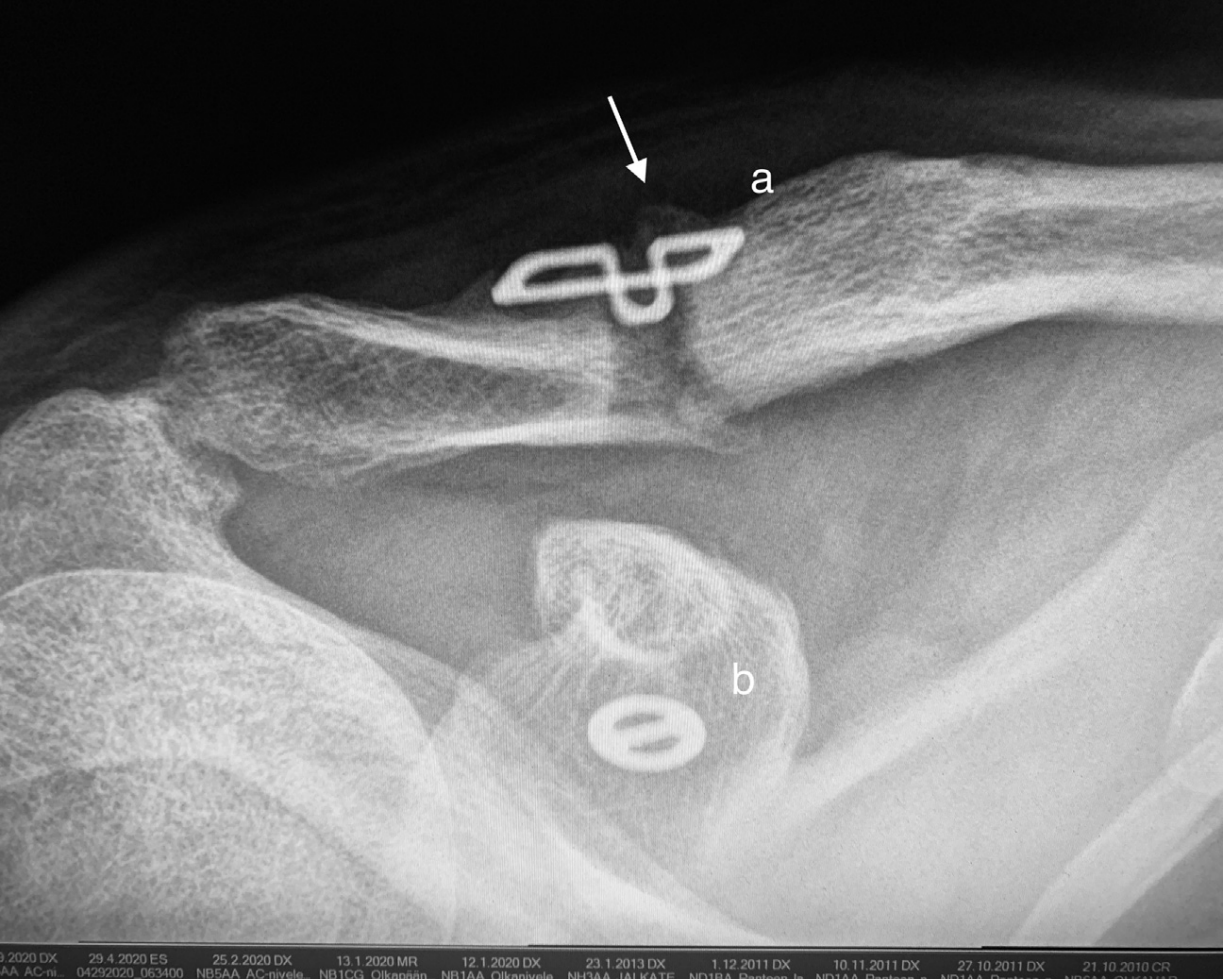
A clavicular fracture occurred in ice-hockey 8 months after the operation. The fracture line went through the clavicular drill hole. The fracture was treated conservatively. The fracture healed, and the tendon reconstruction remained in place. The Clavicular Clip (a), the Subcoracoid Clip (b) is shown. Regenerative bone on the clavicle (arrow). An anteroposterior radiograph of the healed right shoulder 4 months after injury.

There was one unsuccessful reconstruction, which was reoperated 6 months after the original operation. There was one clavicular fracture through the clavicular drill hole. The patient, an ice-hockey player, was rammed against the rink wall affecting the operated shoulder 8 months after the operation. The fracture was in an exact position, and the ligament reconstruction remained unharmed. The situation was treated conservatively, and the fracture healed well. (Fig 3) One AC joint arthroscopic debridement was conducted 1 year after the original operation because of an annoying cracking noise.

No coracoid fractures were found. The obvious risk for a coracoid fracture is transfer of the tendon graft through the 4.5-mm coracoid drill hole. Otherwise, the techniques can be considered similar. Because no coracoid fractures occurred, the groups were handled as one in terms of the postoperative statistics. In seven of the cases, a sudden brief sensation of pain was reported 3–6 months after surgery. It was reported that the distal clavicle felt a little more mobile afterward. The pain disappeared soon, and the clavicle worked normally after that. No other complications occurred in the series. One case was lost from the radiographic follow-up because the patient refused to undergo follow-up radiographs. The reoperated original case was not included in the statistics.

**Fig 4:**
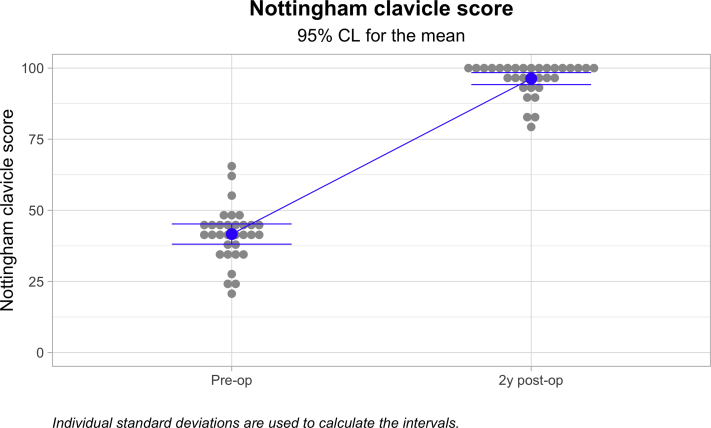
The one-year Nottingham Clavicle scores increased significantly from a preoperative mean of 41.66 ± 9.86 to 96.831 ± 5.86 (*P* ≤ .05).

**Fig 5:**
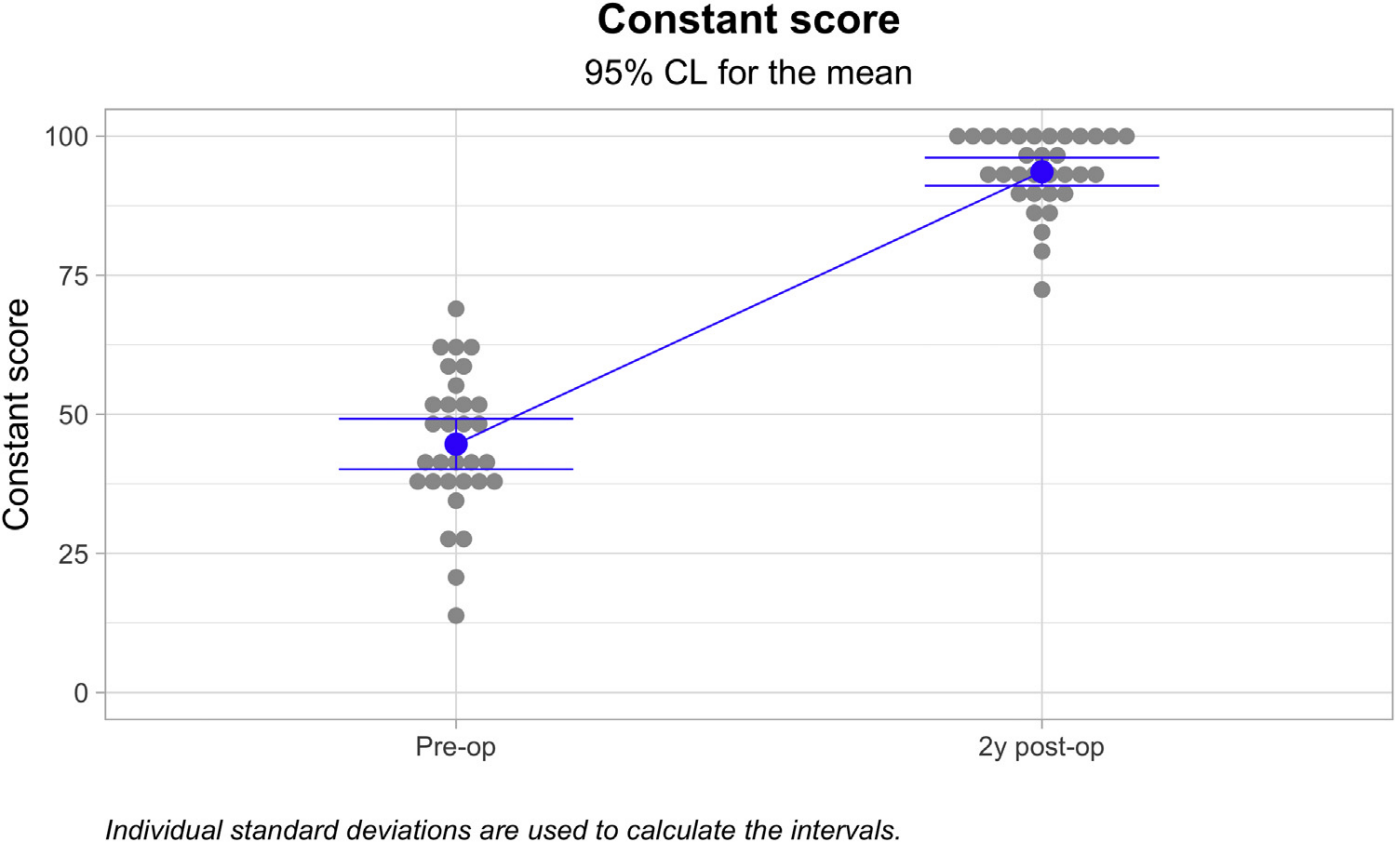
The one-year Constant score increased significantly from a preoperative mean of 44.66 ± 12.54 to 93.59 ± 7.01 (*P* ≤ .05).

**Fig 6:**
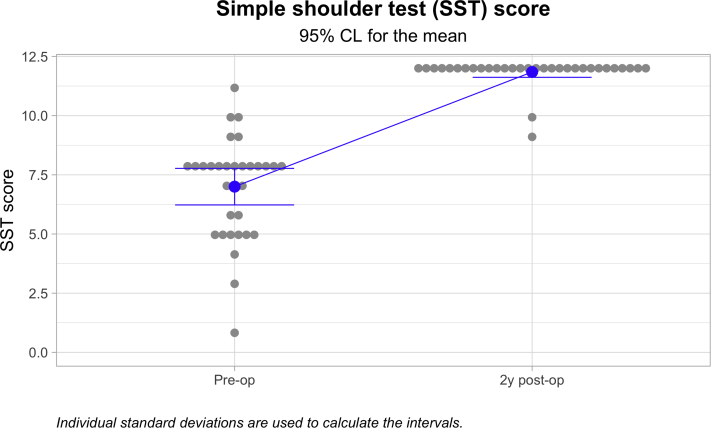
The one-year Simple Shoulder Test score increased significantly from a preoperative mean of 7.00 ± 2.14 to 11.84 ± .63 (*P* ≤ .05).

**Fig 7:**
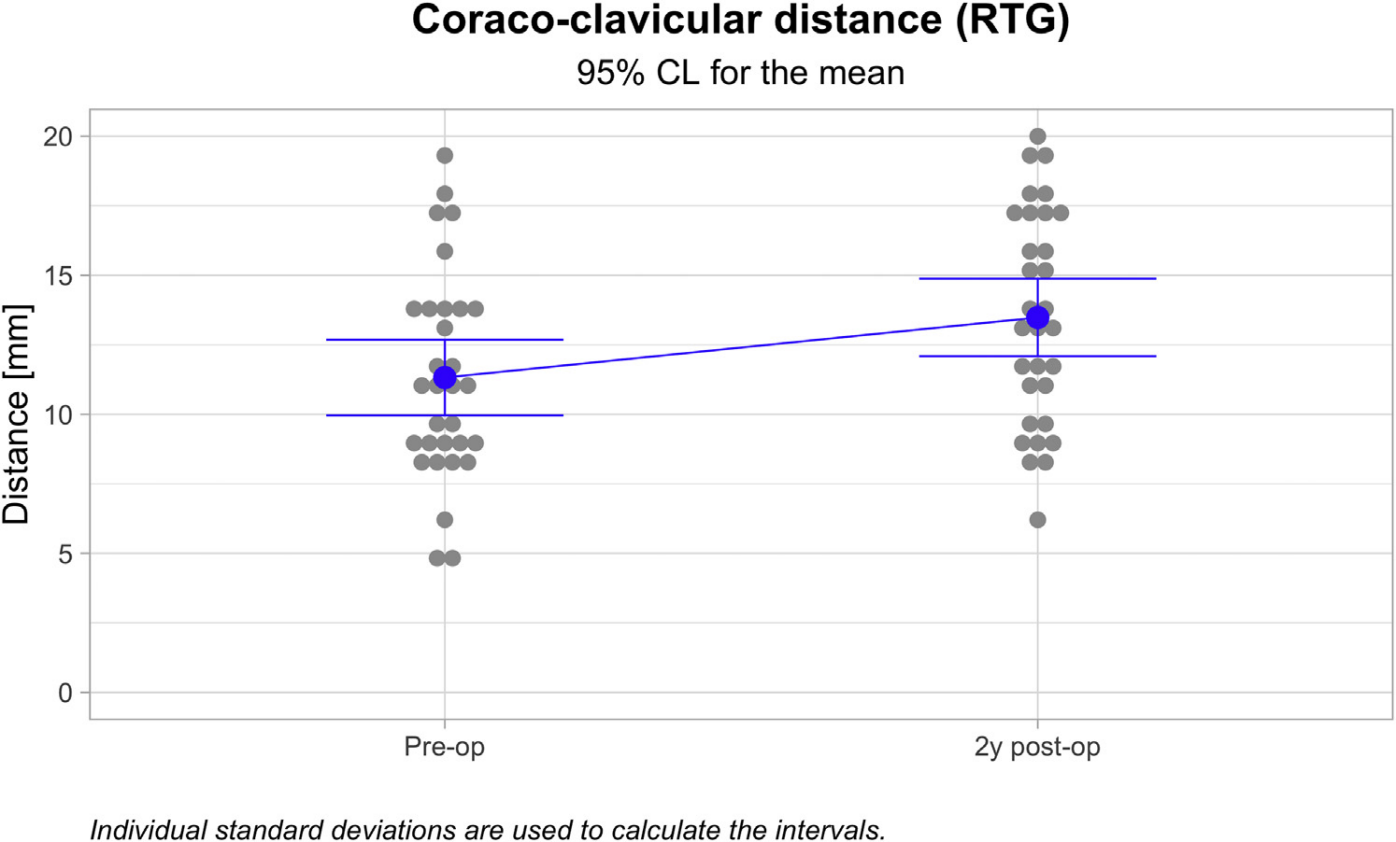
The one-year coracoclavicular distance increased from a preoperative mean of 11.32 ± 3.71 to 13.48 ± 3.79 mm (*P* ≤ .05).

**Fig 8:**
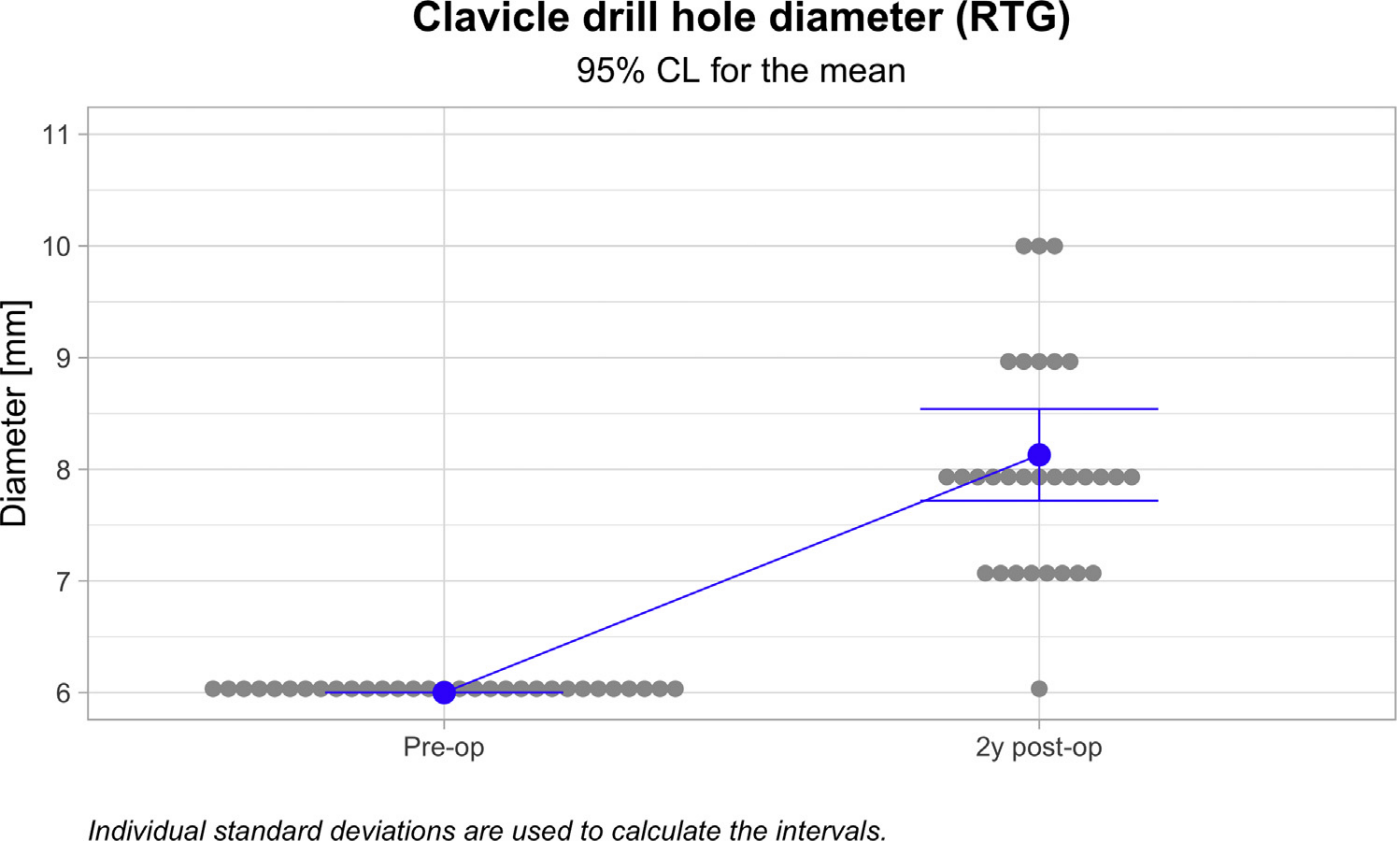
The one-year clavicular drill hole diameter increased from 6 to a mean of 8.13 ± 1.12 mm.

Among the 32 cases followed for a minimum of 1 year, the mean Nottingham Clavicle score increased from a preoperative mean of 41.66 ± 9.86 to 96.831 ± 5.86 (*P* < .000; Fig 4). The Constant score increased from a preoperative mean of 44.66 ± 12.54 to 93.59 ± 7.01 (*P* < .000; Fig 5). The Simple Shoulder Test score increased from a preoperative mean of 7.00 ± 2.14 to 11.84 ± .63 (*P* < .000; Fig 6). The coracoclavicular distance increased from 11.32 ± 3.71 to 13.48 ± 3.79 mm (*P* < .027; Fig 7). The clavicular drill hole diameter increased from 6 to a mean of 8.13 ± 1.12 mm (Fig 8). Patient satisfaction was high. Twenty-three (71.9%) patients reported an excellent outcome, and nine (28.1%) reported a good outcome.

## Discussion

In this study, arthroscopic CC and open AC ligament reconstructions using a tendon graft remained stable, shoulder scores improved, patient satisfaction was high, and no problems occurred in clavicular wound healing. One unsuccessful reconstruction was noted, and the distal clavicle remained pronounced due to too loose tendon reconstruction. Reoperation was successfully conducted 6 months later. Another case needing a repeat operation was due to an annoyingly cracking in the AC joint, in which arthroscopic AC joint debridement was conducted. Interestingly, in the arthroscopy, a torn interconnecting suture was detected. In eight additional cases, a sudden brief sensation of pain was reported 3–6 months after surgery. The distal clavicle felt a little more mobile afterward. These findings led to the notion that after a few months, the interconnecting suture probably fails, and the distal clavicle becomes more mobile once the tendon graft assumes the load. That process may also explain why the distal clavicle appeared slightly elevated during the follow up. The strong no. 5 interconnecting suture applied between the clavicular and subcoracoid clips was considered semitemporary and was expected to eventually snap.

The distal clavicle is stabilized by the CC ligament complex and the AC joint ligaments and capsule.^17^ The CC ligament complex is mainly responsible for vertical stability of the clavicle. The AC joint ligaments and capsule provide anteroposterior stability to the AC joint.^18^, ^19^, ^20^, ^21^

The clavicle is the only rigid, support arm-like connection between the upper extremity and axial skeleton. An unstable clavicle may lead to scapular dyskinesis among other difficulties.^22^, ^23^, ^24^ Therefore, the primary aim of the ligament reconstructions is not to facilitate meeting of the distal clavicle to the acromion again but rather to restore the strong connection between the upper extremity and central skeleton. The need for a tendon graft for achieving a sustainable CC reconstruction is obvious.^25^^,^^26^ It is equally important that the AC joint capsule is assessed.^27^ The reconstruction of the superior acromioclavicular ligament with the graft limb and capsule plication seemed to have improved the stability of the entire reconstruction.^28^, ^29^, ^30^

Another important object of this study was clavicular wound healing. In the previous techniques, wound irritation, infection, and palpable knots on the clavicle were among the problems encountered in open and arthroscopic CC reconstructions.^11^ The Clavicular Clip was designed to be used with a tendon graft and hide protruding suture knots, and therefore, avoid any wound irritations. No wound complications occurred in this series nor could the patients feel any postoperative subcutaneous prominence. In this study, no differences between Groups 1 and 2 were found as no coracoid complications had occurred. In Group 1, the theoretical advantage of taking the graft through both the clavicular and coracoid drill holes is the firm attachment by both ends that the graft obtains into the bone channels, as observed in anterior cruciate ligament (ACL) reconstructions. However, this procedure includes the risk of a coracoid fracture.^31^ In Group 2, the risk of a coracoid fracture is minimal, although the process by which the tendon graft integrates on the coracoid bone surface is unknown. Clearly, tunnel widening is a problem when using both clavicular and coracoid drill holes. Apparently, tunnel widening will occur regardless of whether a tendon graft or plain interconnecting suture is used.^32^ Therefore, the presence of only one clavicular drill hole decreases the risk of a clavicular fracture.

The change in clavicular tunnel diameter was measured using anteroposterior radiographs at minimum of one year after surgery. The measures were obtained half-way to the clavicular drill hole. The anteroposterior radiograph proved to be a fairly unreliable method for assessing the size of the clavicular drill hole. (In two cases, a computed tomography scan control was obtained, and the drill holes were slightly smaller compared to the appearance of radiographs.) Determining the size of a coracoid drill hole proved to be even more difficult. Therefore, tunnel widening of the coracoid drill hole was not measured in this study. The average CC distance increased during the follow-up. The increase was moderate and did not affect the stability of the distal clavicle or the procedural outcomes. The biggest increase was detected in the three revision cases. The reason for this increase may have been the pronounced instability and weaker support for the clavicle by the surrounding muscle attachments. The outcome scores were also slightly inferior. However, all three patients reported good subjective outcomes. The use of the synthetic tendon graft did not seem to affect the outcomes in this series.

The Nottingham clavicle and Constant scores and Simple Shoulder Test improved significantly after surgery. In most cases, the patient satisfaction level mirrored the shoulder score levels. CC ligament reconstructions have been considered as difficult procedures plagued by complications.^33^ However, the results of this study and previous follow-up studies have shown favorable results when using this technique.^12^

An arthroscopic approach in CC ligament reconstruction is a practical method, while the AC ligament reconstruction must be an open procedure. A technical error is usually the most obvious reason for less-than-perfect outcomes. With regard to the surgical technique, it is very important to position the drill holes correctly. The clavicular drill hole must be centrally located. The coracoid drill hole must be located both centrally and as proximally as possible. The distal end of the clavicle must be released from soft tissue interpositions, such as parts of the trapezius muscle, and the distal clavicle repositioning should be tension-free. Distal clavicle resection is recommended in chronic cases. Infection and fracture control and reconstruction stability are the key reconstruction elements. The typical surgical time, including harvesting and preparing the ST tendon, is 1.5–2 hours. It is extremely important that the recovery time is long enough to ensure a successful surgery. Postoperatively, an arm sling is worn for 6 weeks. Physiotherapy is initiated only 8 weeks postsurgery. The tendon graft must heal properly before rehabilitation is started. Therefore, the first 2 months postoperatively are critical. After 2 months, it is unlikely for the reconstruction to fail, excluding new external trauma.

### Limitations

This study is not without limitations. First, we included revision surgeries, which may produce different results than only primary reconstructions. Also, the number of subjects in each group was different. This is because the graft countering the coracoid was used more often, which may also affect the results.

### Conclusions

In this series, combining the arthroscopic CC ligament reconstruction to an open reconstruction of the AC joint with a tendon graft proved to be a stable solution. The knot-hiding titanium implant effectively eliminated the problems related to the clavicular wound healing.

## Supplementary Data

ICMJE author disclosure forms
